# Comparison of the novel Medtentia double helix mitral annuloplasty system with the Carpentier-Edwards Physio annuloplasty ring: morphological and functional long-term outcome in a mitral valve insufficiency sheep model

**DOI:** 10.1186/1749-8090-8-70

**Published:** 2013-04-08

**Authors:** Moritz A Konerding, Jarmo Simpanen, Leo Ihlberg, Juha Aittomäki, Kalervo Werkkala, Vera Delventhal, Maximilian Ackermann

**Affiliations:** 1 Institute of Functional and Clinical Anatomy, University Medical Center of the Johannes Gutenberg-University Mainz, 55099, Mainz, Germany; 2 Department of Cardiothoracic Surgery, Helsinki University Hospital, Helsinki, Finland

**Keywords:** Annuloplasty, Mitral, Valve, Valve insufficiency, Valve repair, Endocardium

## Abstract

**Background:**

The prevalence of mitral regurgitation in cardiac diseases requires annuloplasty systems that can be implanted without excessive patient burden. This study was designed to examine the morphological and functional outcome of a new double helix mitral annuloplasty ring in an ovine model in comparison to the classical Carpentier-Edwards (CE) annuloplasty ring as measured by reduction of mitral regurgitation and tissue integration. The Medtentia annuloplasty ring (MAR) is a helical device that is rotated into the annulus self-restoring the valve geometry, enabling a faster fixation without the need of elaborate repair of the valve geometry. The ventricular part of the helical ring encircles the valve chords.

**Methods:**

Twenty adult sheep were overpaced until 2+ level mitral valve regurgitation was achieved. Seven animals per group received either the MAR or the CE ring. Implantation was performed on-pump in a beating heart through the left atrial appendix. The animals were sacrificed 3.6 ± 0.3 months after surgery following an echocardiography for assessing mitral regurgitation as primary endpoint. The annuloplasty rings with surrounding tissue were harvested for histological analyses as secondary endpoints. The remaining six sheep received the MAR system and were sampled seven, nine or 12 months after surgery.

**Results:**

Implantation time (p < 0.01) and perfusion time (p < 0.001) as clinical secondary endpoints were significantly shorter in the MAR group. Echocardiography follow-ups showed sufficient valve function repair in nearly all animals with a normalization of the ventricle diameters in both groups (group difference: p = 0.147). The weights of the hearts did not differ significantly. Histology revealed adequately covered atrial annuloplasty rings with functional endothelium and lack of excessive granulation tissue or fibrosis in all specimens. The ventricular projections of the MAR systems encircling the chordae tendineae were not completely covered with neointimal tissue, although in no case were microthrombi detected and no thromboembolic events were recorded.

**Conclusions:**

The new MAR system is an easy to use annuloplasty system with a functional outcome comparable to that of the well–proven CE ring. Mitral valve regurgitation is effectively stopped both by restricting the pathological expansion of the annulus and by gathering the chords without thrombus formation.

## Background

The prevalence both of heart valve and cardiovascular disease is expected to increase in the next decades. Recent years have witnessed a clear trend toward a lower level of invasiveness in medical procedures in general, and in cardiac procedures in particular [1]. Ischemic mitral regurgitation (IMR), a consequence of left ventricular dysfunction despite a structurally normal mitral valve, occurs in 19% of patients after myocardial infarction [2]. Chronic IMR is an independent predictor of mortality with a clear correlation to the degree of mitral regurgitation (MR): the greater the degree of MR, the worse the prognosis [3,4].

The repair of mitral and tricuspid incompetence with preservation of the native valve is a major target of modern valve surgery [5,6]. The dilatation of the annulus aggravates regurgitation and thus the implantation of a support prosthesis on the dilated annulus - similar to an annuloplasty ring - is frequently performed to reconstitute its physiological size and shape [5].

**Figure 1:**
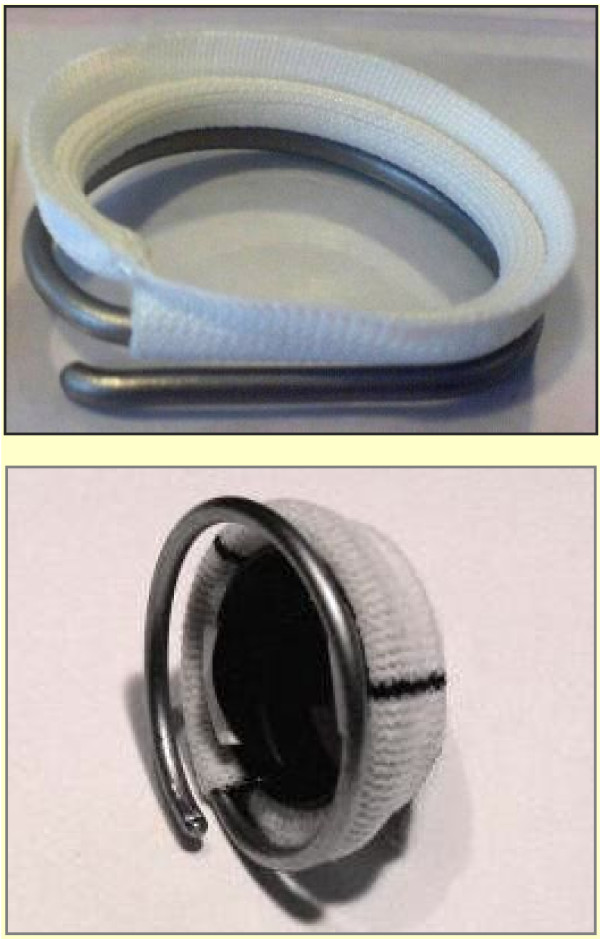
**The Medtentia annuloplasty ring.** The atrial helix arm is covered by a cuff, the ventricular part is uncovered. The carbonated titanium surface effectively prevents cell adhesion and clotting.

To further facilitate faster and easier mitral valve annuloplasty procedures with reduced clamp time, the novel Medtentia double helix mitral annuloplasty ring (MAR) was developed [7]. This ring consists of a double helix similar to a key ring and is implanted by rotating it around the mitral valve leaflets to place one helix arm in the left atrium and the other in the left ventricle (Figure 1). Thus, there is no need for elaborate and time consuming valve repair before fixation of the MAR. This unique feature potentially allows the consideration and development of a variety of different fixation methods that might facilitate easier, minimally invasive approaches. In addition, the ring fixation is potentially more stable compared to rings that are only placed in the atrium, such that the risk of late ring dehiscence may potentially be reduced.

The most critical aspect is that the exposure of annuloplasty rings to blood may induce thrombosis and/or thromboembolism resulting from the adhesion of fibrin and platelets on the artificial tissue and inflammatory reaction [8]. Local healing with foreign body reaction is a normal consequence of synthetic material implantation and leads to the formation of fibrous tissue composed of fibroblasts, foreign body giant cells and extracellular matrix [5].

The aim of this study in mitral insufficient sheep was therefore to elucidate the morphological and functional outcome of the Medtentia double helix mitral annuloplasty ring in comparison to the classical Carpentier-Edwards (CE) Physio Annuloplasty ring in sheep with mitral valve insufficiency.

## Methods

### Animals

In total, 20 female sheep weighing between 46 and 60 kg (mean 51 ± 6 kg) were used for this study. The sheep were obtained from a specialized sheep breeding farm (Kotikoski, Finland), and were kept in group boxes with unlimited access to standard food and water in the central animal facilities of the Veterinary Medicine Faculty of Helsinki University hospital. Surgery, final ultrasound examination, as well as tissue harvesting were performed either in the animal facilities of Helsinki University Hospital or at the Helsinki University Veterinary Medicine Faculty. All experiments were carried out in accordance with the regulations laid down by law after approval of the local institutional review board (approval no. ESLH-2008-008641/m-23 and ESLH-2009-066994/m-23).

### Rapid ventricular pacing

Pacemakers were used to induce mitral valve insufficiency with significant 2+ regurgitation. Pilot studies revealed that higher grades of mitral regurgitation resulted in unacceptably high lethalities for this long term follow-up. In all animals, permanent ventricular pacemakers (Relia S01, Medtronic Finland, Helsinki) were implanted and programmed to a continuous frequency of 170/min. In the first five animals of the series Vitatron C20 pacemakers (Medtronic Finland, Helsinki) were initially implanted, but had to be replaced after failing to induce mitral valve insufficiency. One day to two weeks before the annuloplasty ring implantation, the frequencies were programmed to 140/min in order to ensure survival during the follow-up time.

### Echocardiography

Transthoracic echocardiography (TTE) with assessment of ventricle size and regurgitation was carried out three months after pacemaker implantation. In five cases in which the induction of insufficiency was slower, a further TTE was performed four months after pacemaker implantation. Reference TTEs were obtained during the week prior to surgery. Control examinations were done every four weeks after surgery, as well as immediately before sacrifice. All TTEs were performed blinded by the same investigator (JA) with a portable GE Vivid-I echocardiography system (GE, Helsinki, Finland).

### Study groups and surgical procedure

The sheep were randomly assigned to the individual groups. For the reference group (Carpentier-Edwards Classic, Edwards Lifesciences Nordic AB, Finland), CE annuloplasty rings were implanted into 7 sheep, whereas 13 animals received Medtentia annuloplasty rings (MAR, Medtentia Oy, Helsinki, Finland) with appropriate dimensions. Seven sheep with MAR were intended for the direct comparison with animals with CE after 3.6 months, the remaining six MAR animals were intended for sampling seven, nine or twelve months after surgery.

Surgical procedures were performed under deep anesthesia according to standard procedures. All implantations were done by the same team in order to overcome operator dependency. The sheep were given 5 mg midazolam and 25 mg/kg bw ketamine hydrochloride i.m. as premedication. Anesthesia was induced with intravenous administration of pentobarbital (12 mg/kg bw) and fentanyl (7.5 mg/kg bw).

An endotracheal tube was inserted and mechanical ventilation initiated with tidal volumes set at 8–10 ml/kg body weight with a 100 ml compensation for dead space, at rate of 12–15 cycles per minute (Servo 900C; Siemens Elema AB, Solna, Sweden). The inspired oxygen fraction (O_2_-air) was 0.5 and a positive end-expiratory pressure of 5 cm H_2_O was applied. End tidal carbon dioxide tension was monitored with a capnograph (Datex, Helsinki; Finland). Fentanyl (10 mg/kg/hr) and pentobarbital (5 mg/kg/hr) were given as continuous infusions for maintenance of anesthesia with a mixture of O_2_ and isoflurane. A heating blanket and heater on the cardio pulmonary by-pass system were used to maintain normothermia.

Fluid support (Ringer solution) was delivered intravenously as necessary to maintain the systolic blood pressure above 80 mmHg. For continuous monitoring, the left femoral artery was cannulated for systemic arterial pressure monitoring. The ECG and oxygenation were continuously monitored (Datex). After thoracotomy and administration of heparin (400 IU/kg), the carotid artery was cannulated with a wire-forced cannula (18 F) for cardiopulmonary bypass (CPB). The 2-stage venous cannula (22 F) was cannulated for the drainage of the venous return into a cardiotomy reservoir with a pediatric hollow fiber oxygenator (Jostra, Hirrlingen, Germany). A Gambro roller pump (Gambro, Lund, Sweden) was used for CPB with a mixture of heparinized Ringer acetate (50%) and blood from donor sheep (50%) as the priming solution. The oxygenator was heated with a water coil (Heaterâ, Tekamer, Helsinki, Finland). The mitral annuloplasty ring implantation was performed on-pump in a beating heart through the left atrial appendix.

The MAR ring was rotated into place starting at the posterior medial commissurae. The MAR was rotated 360 degrees so that the lower ring of the MAR could slide onto the ventricular aspect of the annulus, behind and underneath all chordae. The ring was fixed with a total of 6 to 10 2–0 pledgeted Ethibond sutures that were placed in a classical manner to the valve annulus and through the Dacron cover on the atrial aspect of the ring. The sutures’ placements were indicated in the surgery protocol of the case report forms. Reference CE rings were implanted according to the manufacturer’s instructions with sutures similar to those used for the MAR rings.

### Post-operative care and anticoagulation treatment

After surgery, animals were transported back to the animal facility for follow-up. Antithrombotic treatment consisted of 5.000 IU dalteparin s.c. (Fragmin) for the first 3 days. Diclofenac 75 mg (Voltaren) was given b.i.d. for the first 5 days after surgery and thereafter as necessary. The antibiotics ceftriaxone (Rocephalin; 1.5 g o.d.) and tobramycin (Tomycin; 120 mg o.d.) were given prophylactically for 5 and 3 days, respectively.

### Sampling

The animals of the CE group and seven animals of the MAR group were sacrificed after 3.6 ± 0.3 months follow-up time. Of the remaining MAR group animals, two were sacrificed at seven, nine, and twelve months after annuloplasty.

On the day of sacrifice or one day before, animals were transported to Helsinki University Hospital or the Helsinki University Veterinary Medicine Faculty, where they were sedated with 5 mg midazolam and 25 mg/kg ketamine hydrochloride i.m. as premedication and euthanized with propofol and an overdose of 40 ml KCl administered intravenously through a cephalic vein catheter.

The thoracic cavity was opened and the heart freed by dissection. After flushing and weighing, the transverse and longitudinal dimensions were assessed with a ruler. Blocks of the myocardium were sampled in defined areas of the ventral and dorsal aspects of the ventricles and atria. The annuloplasty rings were dissected free and 2 × 1.5 mm areas of the neointimal layer were harvested using a scalpel.

### Histology

The myocardial samples were embedded for light microscopy using a standard protocol and stained with H&E. Cell densities and proliferation were assessed in the neointimal tissue probes using an anti-PCNA monoclonal antibody. Quantification was performed using image analyzing software (Diskus 4.80; Hilgers, Königswinter, Germany). All sections underwent blinded quantitation by an independent observer.

Vessel ingrowth and neointimal surface classification was carried out using an anti-FVIII antibody. Both antibodies were purchased at BD Biosciences Pharmingen (Heidelberg, Germany). Antibody binding was visualized via a three-step staining procedure using a biotinylated polyclonal anti-rat Ig-G secondary antibody (DakoCytomation GmbH, Hamburg, Germany) and the streptavidin horse-radish peroxidase reaction together with the DAB detection system.

The dissected, implanted rings were cut into two halves, one of which was embedded in methylmetacrylate (MMA), and the other processed for scanning electron microscopy.

The integration of the implanted rings was evaluated in polished microsections of MMA-embedded specimens that were cut into 500–800 μm thick sections with a diamond blade saw and grinded down to 30–70 μm between rotating glass plates covered with 1200 grade wet sand paper. Specimens were stained en bloc with H&E; the sections were documented with a Zeiss Axiophot microscope (Oberkochen, Germany).

The remaining half of each annuloplasty ring was used for scanning electron microscopic analysis of the surface structure. The specimens were fixed in glutaraldehyde, trimmed, rinsed in phosphate buffer, postfixed in osmium tetroxide, rinsed again and placed in acetone solutions of ascending concentrations. After drying, the rings were mounted on specimen holders and sputtered with gold in an argon atmosphere prior to scanning electron microscopy (Philips ESEM XL 30; FEI, Eindhoven, Netherlands).

### Statistical analysis

Data were expressed as mean ± standard deviation. One-way analysis of variance (ANOVA) and Student’s *t*-test for unpaired data were used for differences between groups. The difference was considered statistically significant at a level of p < 0.05.

## Results

### Induction of mitral valve insufficiency

**Figure 2:**
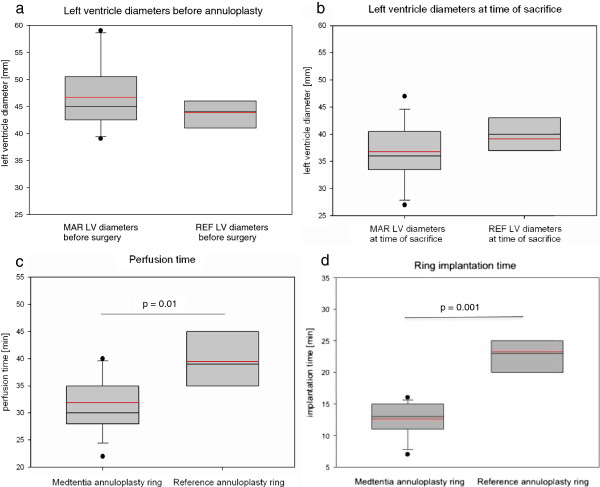
**Clinical parameters: Left ventricle diameters assessed by echocardiography before annuloplasty (a) and at the time of sacrifice (b). c, perfusion time, and d, ring implantation time.** Boxplots with 25, 50 and 75th percentiles. Red line = mean.

The time span needed for induction of a significant mitral valve insufficiency with positive regurgitation was 140 ± 31 days in the animals designated for the MAR and 117 ± 29 days in the animals designated to the CE group. The TTEs prior to implantation showed a 2+ level of regurgitation in all but one animal in the MAR group and one animal in the control group, both of which had only a 1+ level of regurgitation. Figure 2a shows the diameters of the left ventricle at the papillary muscle level, which, in the MAR group had a mean diameter of 46.7 ± 6.50 mm, comparable to the mean diameter found in the CE group (43.9 ± 3.39 mm).

### Implantation time, survival and side effects

All annuloplasty rings were implanted successfully. Intraoperative water tests revealed competent and tight valves in all animals. Postoperative recovery was uneventful. The time needed for surgery was shorter in the MAR group (81.9 ± 16.7 min) than in the CE group (94.2 ± 24.4 min). The perfusion time in the MAR group (31.8 ± 5 min) was also significantly shorter than in the CE group (39.4 ± 6.2 min; p = 0.01; Figure 2c). The total annuloplasty ring implantation time of 12.6 ± 2.6 min in the MAR group was again significantly shorter than that needed in the CE group (23.4 ± 4.2 min; p = 0.001; Figure 2d).

During the follow-up time, one animal in the MAR test group was found dead 350 days after surgery, shortly before completion of the one year follow-up. The TTE in this animal, performed eight weeks after surgery, showed no regurgitation, whereas moderate-severe regurgitation was seen 6 months after surgery. The left ventricular end-diastolic diameter was found to have increased to 47 mm. Autopsy revealed – besides the accumulation of 7–8 liters ascites - that the annuloplasty ring had not caught hold of all chordates, which explains the reappearance of regurgitation. All other animals reached the designated end point without complications.

### Echocardiography

TTE prior to tissue sampling revealed no regurgitation in eleven MAR-test animals and in all control animals. One test animal showed 2+ level regurgitation and another animal revealed a mild-moderate regurgitation one year after surgery. The half-year echocardiographic examination of this animal showed no signs of valve insufficiency. The end-diastolic left ventricle diameters (Figure 2b) showed no significant differences between the MAR group (36.8 ± 5.43 mm) and the CE group (39.1 ± 4.18 mm; p = 0.147).

### Heart weights

The echocardiography data was analogous to the data on heart weights: after 3.6 months follow up, the CE group’s mean heart weight (290.0 ± 41.2 g) was comparable to that of the MAR group (297.3 ± 46.3 g; p = 0.38). The same was true when the slightly heavier hearts of the long-term follow–up animals (314.6 ± 33.4 g) were compared to those of the short term animals (p = 0.235).

### Macroscopic findings

**Figure 3:**
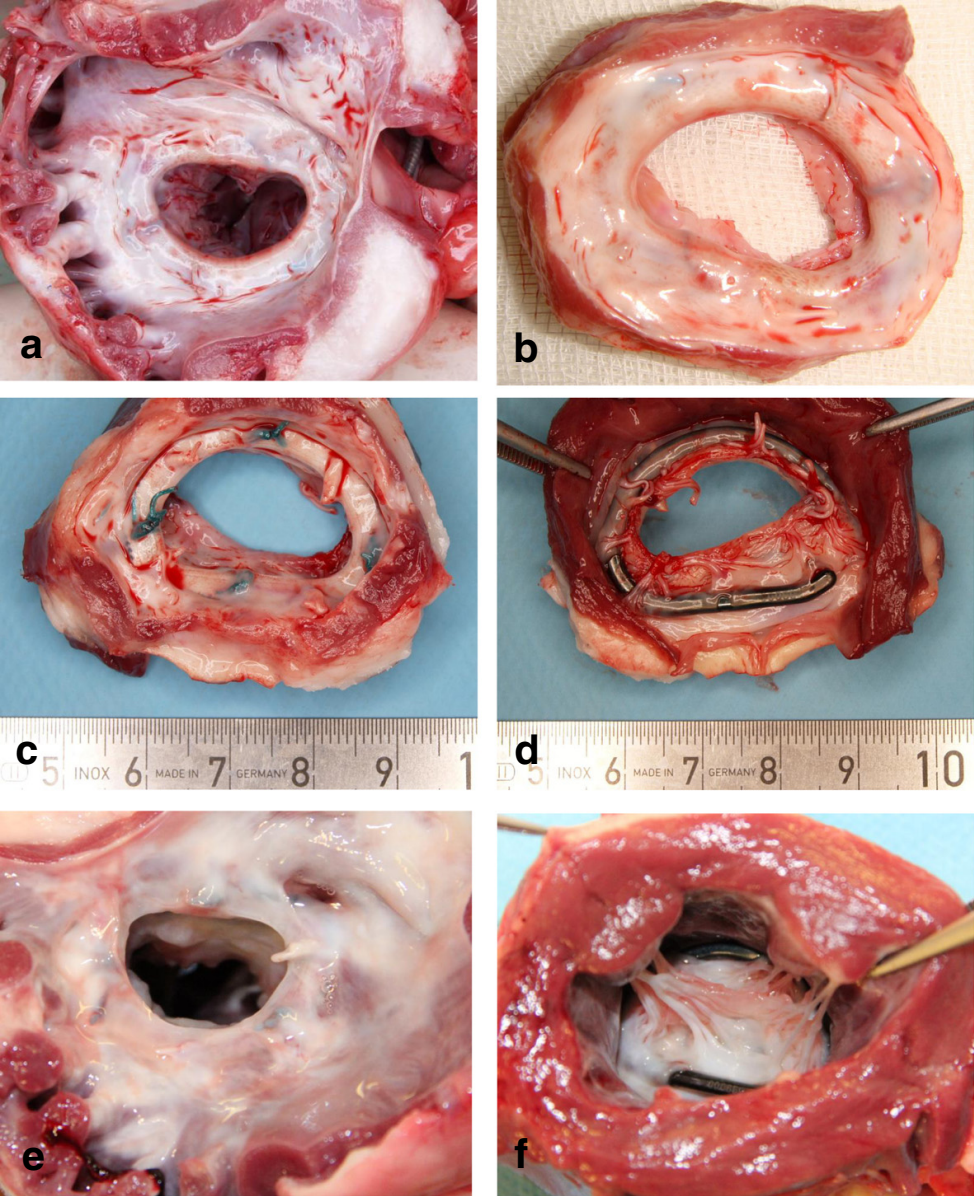
**Annuloplasty rings after sacrifice. a**: Edwards-Carpentier annuloplasty ring seen from the atrial side 16 weeks after implantation. **b**: shows an example 14 weeks after implantation. Note the macroscopically complete coverage of the device with connective tissue and the absence of thrombi. **c** and **d** show the Medtentia annuloplasty ring 16 weeks after implantation. Note that the ventricular aspect is only partially covered with endocardial tissue. **e** and **f**: Medtentia annuloplasty ring nine months after harvesting from the atrial (e) and ventricular aspect. Note the partially missing coverage from the ventricular aspect.

As shown in Figure 3, all reference group implants were macroscopically completely covered with connective tissue. The shiny, reflecting surface suggested a reasonable coverage with endothelial cells. No thrombi were visible.

The atrial side of all Medtentia annuloplasty rings (Figure 3c and d) also appeared to be completely covered with whitish, shiny connective tissue as a neoendocardial layer. The coverage of the ventricular part of the devices screwed around and caging the chordae tendineae varied between 5-50%. Some bridges of subendocardial tissues were visible; the remaining surfaces were directly exposed to the circulating blood. However, in no case were thrombi present. Almost complete neoendocardial coverage of the ventricular part was only seen after 3.6 months in one sheep. Additionally, in animals sacrificed after longer follow-up, i.e. after 7, 9, or 12 months after surgery, a complete neoendocardial layer was seen on the atrial side together with limited coverage on the ventricular side (Figure 3e, f). Again, in no case were thrombi detectable.

### Microscopic findings

**Figure 4:**
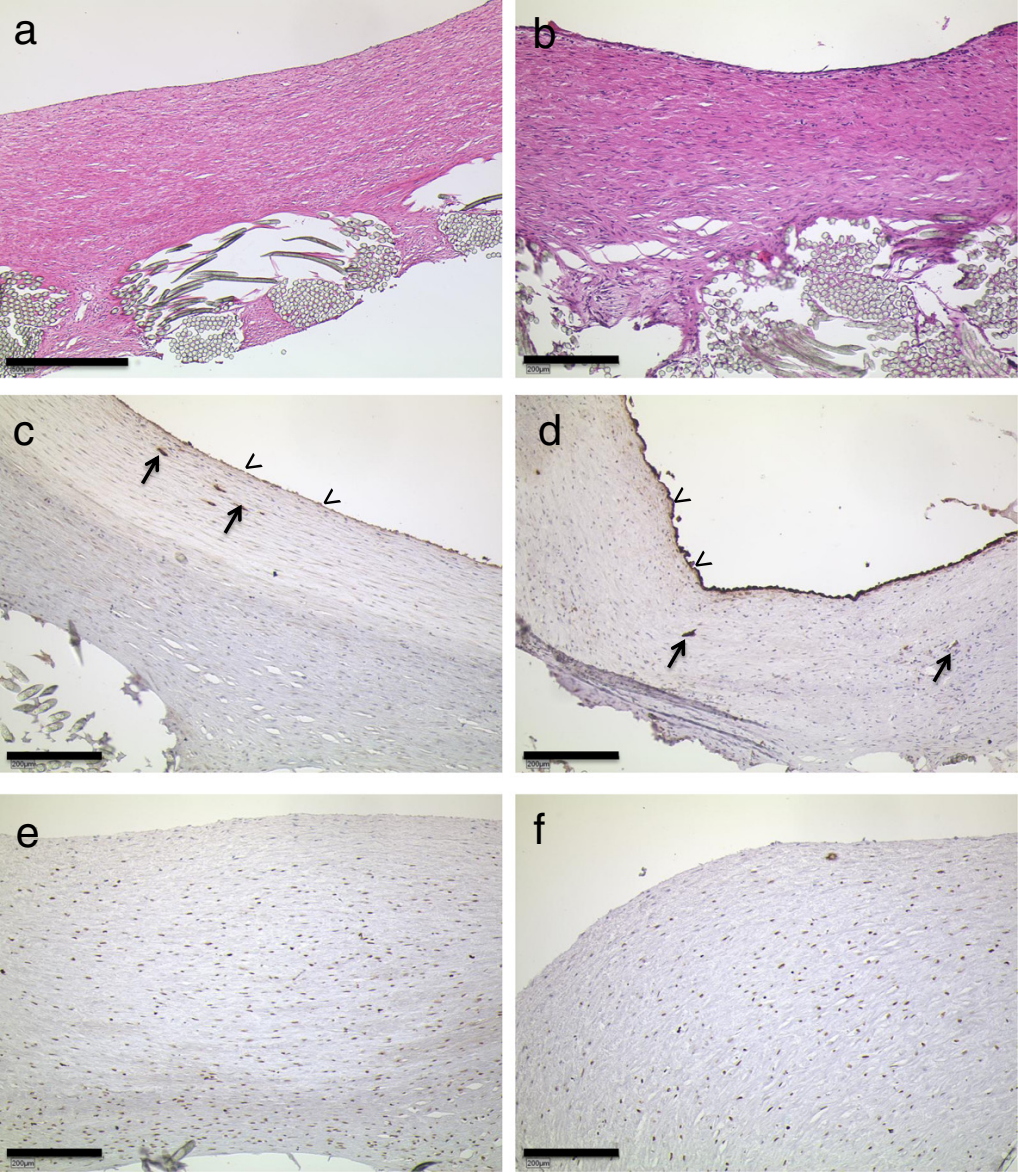
**Microscopic findings in reference annuloplasty rings (left panel, a, c, e; and Medtentia annuloplasty ring (right panel, b, d, f). a & b: H&E histology of the newly formed endocardial tissue.** Note the significantly thicker layer in the reference sample (bar = 500 μm) than in the Medtentia annuloplasty ring (bar = 200 μm). **c** &**d**: anti-Factor VIII staining reveals a functional endothelium on the surface (arrowheads) and some subendocardial vessels (arrows). **e** &**f**: anti PCNA stains show the fraction of mitotic and postmitotic cells with brownish nuclei. Bars in b-f = 200 μm.

The neoendocardial tissue consisted of varying amounts of subendothelial, fiber-rich connective tissue (Figure 4). The suture material was well integrated into this tissue, with some foreign body reaction with multinucleated and histiocytic cells, and the beginning of small granuloma formation being visible in places.

The surface of this newly formed subendocardial tissue was covered with flatly extended endothelial cells that were positive for anti-FVIII (Figure 4c, d). Within the neointima, numerous newly formed vessels were also seen with a functional endothelium and signs of perfusion.

The cell packing densities appeared to be different between the MAR test specimens and the reference group specimens (Figure 4e, f). However, morphometrical assessment of the total cell densities and the PCNA-antigen positive cells (marker of proliferation) revealed both in the superficial, subendothelial layer and the complete tissue layer no significantly different cell and proliferation densities (data not shown).

### Implant integration

**Figure 5:**
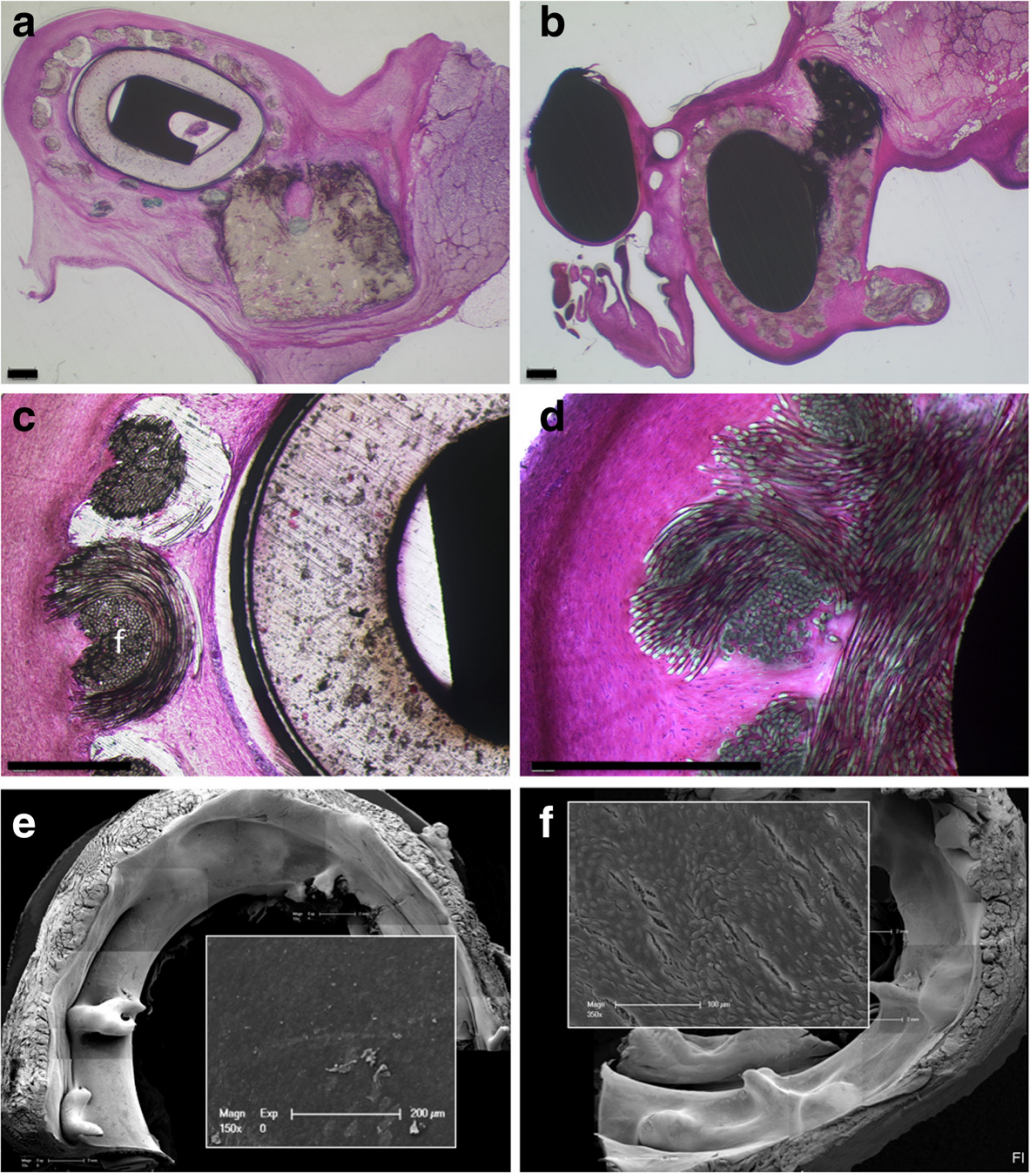
**Implant integration as seen in polished MMA microsections and scanning electron microscopy.** Left panel from reference CE animal 16 weeks after implantation, right images from MAR animal. The coating meshes of both annuloplasty rings are fully penetrated by cells and connective tissue. The thicknesses of the layer seem to be larger in the reference. In **b**, the atrial ring is to the right, the ventricular part is not completely covered with newly formed tissue. **d** shows details of the sleeve integration. The insets in **e** and **f** show the complete endothelial coverage. Bars in a-d = 500 μm.

Apart from the lower, ventricular part of the MAR-specimens, practically all annuloplasty rings showed very good tissue integration (Figure 5). The coating mesh was fully penetrated by cells and connective tissue in all specimens of both groups. The foreign body reaction was comparatively low; in some regions a few foreign body giant cells could be seen. In only one case were sites with greater amounts of granulocytes detectable. The tissue surrounding the implant was seen to contain many small vessels.

### Scanning electron microscopy

Annuloplasty rings with well endothelialized areas were mainly seen at protected sites, e.g., under sutures or lateral to the main blood stream (Figure 5c, d). Other parts showed disrupted or missing endothelium with exposed collagen fiber bundles, especially at the ring angles. These findings are consistent with those seen with factor VIII staining. Qualitative or quantitative differences in the surface structures of the specimens of the different groups were not found.

## Discussion

The percutaneous management of valvular heart disease has recently received a great deal of interest and is considered to be an area of great potential. Innovative technologies are now being developed to treat mitral regurgitation [9] in order to meet the need for less complex, reproducible and fast repair techniques which may be added without further time delay to other heart procedures. The Medtentia annuloplasty double helix system may further facilitate the treatment of mitral regurgitation. The aim of this study was to evaluate this system in an ovine model in comparison to the CE annuloplasty ring. The superpaced animals used for this study had significantly enlarged left ventricle diameters and moderate mitral valve regurgitation. A pilot test on six animals without annuloplasty revealed that cessation of the superpacing after induction of mitral valve insufficiency did not result in spontaneous left ventricle diameter reduction and/or reduction of the regurgitation within six months observation time (data not shown).

This ovine study has demonstrated that the MAR system may be implanted in a significantly shorter time than the well-proven CE annuloplasty ring enabling a shortening of additional clamp time in critical or combined disease patients. This is made possible by the helical construction principle that connects atrial and ventricular surface of the annulus and which restores the valve geometry by screwing in the double helical device. Contrary to other annuloplasty systems the MAR needs stiches only for for fixation, while in conventional mitral rings the sewing of the annulus to the ring prosthesis is the active, more time consuming valve geometry repair step.

The efficacy in terms of functional outcome - as assessed using echocardiography - proved to be in the same range as that of the reference group. In one animal, that was mitral valve competent at the 3 months follow-up echo, the situation at six and nine months was seen to have worsened. Autopsy revealed that the ventricular helix part had not correctly encircled all tendinous cords underlining the mode of action of the MAR system, namely the narrowing and stabilizing of the valve entrance and guidance of the chordae tendineae. The latter shows similarities to the concept of the Alfieri stitch [10].

Material biocompatibility and hemocompatibility are of utmost importance both for tissue integration, avoidance of fibrosis, thrombosis and neoendocardialisation. Practically all artificial surfaces are capable of activating platelets and coagulation factors, which may result clinically in thromboembolic complications. The surface structure of the MAR system with its carbon coating, however, prevented the formation of microclots in areas that were not covered with neointimal tissue during the follow-up. Clinically, no thromboembolic events were recorded even though the animals received no anticoagulative treatment. Della Barbara et al. [6] reported similar results in a pyrolytic carbon-coated flexible Sovering annuloplasty ring.

The atrial sides of both MAR and reference annuloplasty rings were always completely integrated with fibrous tissue covered with a continuous layer of endothelial cells. The absence of complete coverage in the ventricular projections may be due to the hemodynamics: the high pressure and flow gradients as well as the direct contact of the chordae tendinea with the material induce high shear stress that may effectively prevent cell adhesion. The absence of microthrombi, however, indicates that this is not a major shortcoming. The question is, of course, whether this may be extrapolated to the human situation. Several studies have favored the sheep model as an acceptable animal model for testing the blood compatibility of devices [11]. In a study performed in the early 1970s, coagulation values were compared between mature sheep and normal human adults [12]. The significant differences found which were related to the hemostatic system were a markedly decreased fibrinolytic activity and an increased platelet number and adhesiveness in sheep. These factors could be responsible for the increased tendency toward hypercoagulability.

## Conclusion

Both clinical and histological parameters show that the MAR system is a promising new tool for simple and effective treatment of mitral regurgitation.

## Abbreviations

CE: Carpentier-Edwards physio annuloplasty ring; MAR: Medtentia double helix annuloplasty ring; TTE: Transthoracic echocardiography; MR: Mitral regurgitation; CPB: Cardiopulmonary bypass; MMA: Methyl metacrylate; H&E: Hematoxylin-Eosin; PCNA: Proliferating cell nuclear antigen.

## Competing interests

The authors disclose a research grant from Medtentia Oy, Helsinki, Finland, covering all material costs for this study.

## Authors’ contribution

MAK, LI, JS, and KW participated in the conception and design of the study. Animal surgery was performed by JS, LI and KW. All echocardiographies were carried out by JA. Tissue harvesting, histology, electron microcoscopy, and mormometry as well as statistics were done by MAK, VD, and MA. MAK drafted the manuscript. All authors have read and approved the final manuscript.
